# An Inverse Analysis of Interfacial Parameter Values for Mode I Debonding Between Steel and Hot-Melt Adhesive

**DOI:** 10.3390/ma18204648

**Published:** 2025-10-10

**Authors:** Jun Shi, Jian Zhang, Mingzhen Hu, Yingjie Li, Guide Deng, Wenjun Liu

**Affiliations:** 1 Key Laboratory of Special Equipment Safety and Energy-Saving for State Market Regulation, China Special Equipment Inspection & Research Institute, Beijing 100029, China; 2 Hubei Provincial Key Laboratory of Chemical Equipment Intensification and Intrinsic Safety, School of Mechanical and Electrical Engineering, Wuhan Institute of Technology, Wuhan 430074, China; 3 Hubei Provincial Engineering Technology Research Center of Green Chemical Equipment, School of Mechanical and Electrical Engineering, Wuhan Institute of Technology, Wuhan 430205, China; 4 Anhui Tongguan (Lujiang) Mining Co., Ltd., Hefei 231561, China; 5 Hubei Xingxin Technology Co., Ltd., Ezhou 436001, China

**Keywords:** double cantilever beam, interfacial debonding, cohesive zone model, inversion calculation, crack propagation

## Abstract

A polyethylene pipe reinforced with winding steel wires (PSP) is a new composite pipe in which steel wires are effectively bonded with high-density polyethylene (HDPE) through hot-melt adhesive, ensuring the mechanical properties and structural integrity of the pipe. One of the main failure modes at the PSP joint is the interfacial debonding between the steel wire and the hot-melt adhesive. To find a good method to overcome this debonding failure mode, the first priority is to be able to quantitatively characterize the interface performance. Thus, in this study, double cantilever beam (DCB) tests are used to investigate the interfacial properties between steel and hot-melt adhesive, and a finite element model with cohesive element representing the adhesive interface is established to analyze the interfacial properties and the interfacial failure process. However, the interfacial parameters, including interface strength and fracture energy, cannot be obtained directly; thus, based on the inverse optimization calculation concept, an ABAQUS–Python–MATLAB interactive program is developed to continuously optimize and adjust the key parameters of the interface during iterative calculations so that the load–displacement simulation curve is close to the experimental curve, thereby determining the solution set of interface strength and fracture energy. With the inversion parameters substituted into the DCB model, the simulated reaction force–displacement curve is obtained, and it is consistent with the experimental one. Furthermore, this paper compares the pattern of simulated crack tip propagation during the loading process with the experimental results, and it is found that the simulated curve agrees well with the trends of the experimental ones. This proves the effectiveness of the DCB finite element model and the inversion calculation method from a new perspective, indicating that the simulation results of the DCB model were consistent with the experiment. This method can provide guidance and reference for the mechanical behavior analysis of the bonding interface of other materials or structures.

## 1. Introduction

**Figure 1:**
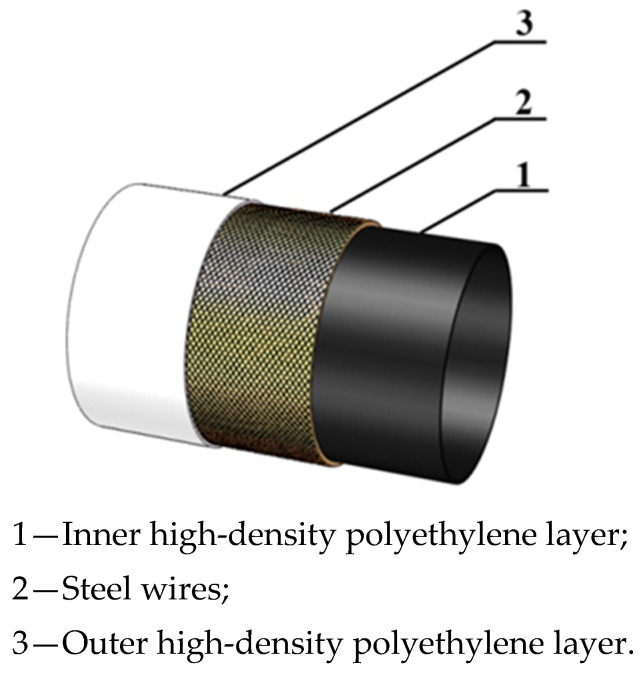
Schematic diagram of PSP structure.

Polymer-based composite materials are widely used in aerospace, automotive, and electronic equipment industries due to their excellent mechanical properties, low cost, and design flexibility [1]. Fiber-reinforced composites with different properties obtained by using various forms of fiber as reinforcement have gradually replaced traditional materials [2]. Polyethylene pipes reinforced with winding steel wires (PSP) have become one of the main alternatives to steel pipes due to their good load-bearing capacity, excellent corrosion resistance, and low maintenance costs [3]. PSP is mainly composed of steel wire, high-density polyethylene (HDPE), and hot-melt adhesive. As shown in Figure 1, the steel wire is helically wound along the axial direction on the outer surface of the high-density polyethylene core. The high-strength steel wire is bonded to the high-density polyethylene by hot-melt adhesive to form an integral structure, and, finally, high-density polyethylene is extruded again as the protective layer of PSP [4,5].

**Figure 2:**
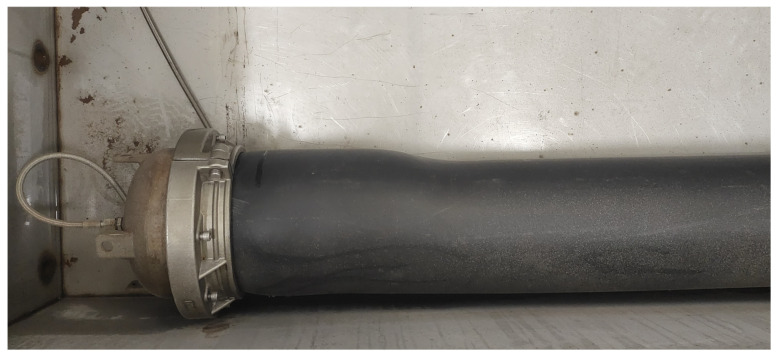
The bulging failure in PSP ends.

In recent years, many scholars have conducted research on the mechanical behavior of the PSP under various loadings, including internal pressure, high temperature, bending moment, radial compression, and related mixed loading [3,4,5,6,7,8,9,10]. Investigation of the steel/hot-melt adhesive interface is rare, but the interface plays a crucial role in the stress transfer between fibers and the matrix, directly affecting the mechanical properties of fiber-reinforced composite materials [10]. With the increase in the load on composite materials, the possibility of excessive bulging deformation in the PSP ends is increasing, as shown in Figure 2.

Based on our early research [9,10], through groups of PSP joint hydrostatic tests, it was found that the interfacial debonding between the steel wires and the adhesive is the main factor causing the bulging failure. Thus, the cause and the process of the interfacial debonding between the steel wires and the adhesive are research priorities [10]. There are various methods to characterize the interfacial debonding behavior, such as the extended finite element method (XFEM), the Virtual Crack-Closure Technique (VCCT), the cohesive zone model, etc. [11]. Xie et al. [12] combined XFEM and experiments to study SiCO thin-film/substrate fracture and interfacial strength, fitting XFEM-calculated P-h curves with nano-indentation results to obtain interface properties and predict critical loads via scratch simulations. Nakrani et al. [13] used XFEM to explore fatigue crack growth at a WAAM-deposited SS316L/SS316 interface, revealing crack deflection driven by stress inhomogeneity and aligning with experiments. Zhong et al. [14] applied XFEM to shale hydraulic fracturing, modeling multi-weak interfaces with unified enrichment functions to evaluate stress intensity factors and predict fracture–interface interaction. Through this literature review, it can be found that the XFEM is capable of modeling complex crack patterns and interfaces with complex behavior, and it enables simulations with reduced mesh distortion and mitigates mesh dependency issues. However, the XFEM exhibits higher computational expenses compared to traditional FEM, and numerical stability can be challenging for highly nonlinear problems.

Teimouri F [15] investigated assessing failure responses of microstructures across several cases of short fiber inclusions. Comparisons between the VCCT and XFEM-VCCT methods were conducted using 2D and 3D finite element simulations of fatigue delamination growth within DCB composite specimens under high-cycle loading. The VCCT model was found to offer a good compromise between high accuracy and low computational cost for 2D and 3D shell models. Karmakov S [16] conducted a critical investigation of the advantages and disadvantages of employing VCCT and XFEM to predict the delamination of carbon FRP composite laminates in distinct fracture scenarios. Delamination was evaluated using simulations of three typical tests: DCB, end-notch flexure (ENF), and mixed-mode bending (MMB). It was found that the VCCT is efficient for modeling crack growth, but it is limited to small deformations and linear elasticity, and it requires a predefined crack path. It also requires relatively high computational costs.

The cohesive zone model is one of the most widely used models [17,18,19,20]. It simulates the damage process of the interface by describing the traction–separation law [21], and it can predict the initiation and propagation of cracks quite efficiently. However, the interface parameters of the cohesive zone model are difficult to obtain, including interfacial strength and fracture energy [22]. There have been a few research papers on determining cohesive parameters through experimental methods, such as the double cantilever beam test (DCB) and end-notch flexure (ENF), which are widely used to test interface fracture energy [11]. However, these methods have high requirements for experimental measurement techniques and equipment [23,24,25]. Due to the limitations of experiments and the complexity of interfacial fracture, many scholars adopt inverse analysis methods to obtain interfacial parameters of the cohesive zone model [26,27,28]. Inverse analysis includes three parts: actual experiments, numerical simulations, and optimization algorithms [29]. Kang [30] proposed an inverse analysis method based on genetic algorithms (GAs) to obtain the interface parameters of metal-based composite material Al/Al_2_O_3_. Zhou [29] used an inverse method based on a pattern search algorithm to study the interface fracture energy of the adhesive in a large-scale yielding (LSY) phenomenon. However, some scholars have found that for inverse algorithms, the uniqueness of the solution is an important issue that needs to be addressed [31,32,33]. Xu [26] combined the Kalman filter algorithm (KFA) with genetic algorithms (GAs), utilizing KFA to enhance the efficiency of solving and GA to enhance the accuracy of inverse analysis. Weighting was allocated according to different situations to obtain a unique solution quickly. Tariq et al. [34] proposed a machine-learning-aided inverse parameter identification framework integrating FEA, optimization, and models like Artificial Neural Networks (ANNs). It successfully calibrated eight cohesive zone model (CZM) parameters, four hardening model parameters, and five Johnson–Cook parameters, achieving excellent simulation–experiment fit and 2–3x faster efficiency than traditional algorithms. Papa et al. [35] developed a robust inverse procedure for cyclic CZM parameter identification in fatigue crack propagation. Using a meta-model to replace FEA and Monte Carlo methods, it utilized fatigue crack growth curves and surface deformation data, providing guidance on minimum measurements for well-posed inverse problems. Gaynutdinova et al. [36] applied a Bayesian approach with the Metropolis–Hastings Algorithm (MHA) to estimate micromechanical moduli of fiber-reinforced composites via Integrated Digital Image Correlation (IDIC). Though computationally costlier than deterministic IDIC, MHA better handles boundary noise, optimizes more parameters, and yields statistical insights, with a non-normalized approach enhancing robustness. It can be found that various inverse calculation methods have been widely used in the determination of the interfacial cohesive parameters.

This paper proposed an effective inverse method to obtain the interfacial parameters between the steel and the hot-melt adhesive. Firstly, the material properties of the steel and the holt-melt adhesive were obtained, and double cantilever beam (DCB) specimens were prepared to conduct mode I interfacial failure for acquiring the load–displacement curves. However, the test data from the DCB interfacial failure could only reflect the overall load-bearing capacity of the adhesive interface, and they failed to characterize the interfacial mechanical behavior and explain the interfacial failure process in detail. It was also difficult to provide effective interfacial failure criteria for structures in practical applications. Therefore, a finite element model consistent with the double cantilever beam specimens was established with ABAQUS. Furthermore, an ABAQUS (2019 version)–Python (2.7 version)–MATLAB (2022 version) interactive program was developed to perform an inverse calculation that generally implements an iterative loop calculation function. As for each round of iteration, the input values of the interfacial parameters for the finite element model were different and adjusted automatically using the pattern search algorithm. The purpose of this inversion program was to make the calculated result curve increasingly close to the experimental result curve after multiple rounds of iteration. Based on the developed program, interfacial parameters were determined. Furthermore, the propagation process of the crack tip with loading displacement was reproduced by substituting the parameters obtained from inversion into the finite element model. The comparison of the crack tip propagation was conducted between the simulated and experimental results to verify the inversion method.

## 2. Experimental Section

### 2.1. Specimen Preparation

In terms of material selection, 45# steel was used for the metal substrate, which was a potential material that could be used as the reinforcement in the PSP. The hot-melt adhesive was produced by Qingdao Koyo Polymer Materials Co., Ltd. (Qingdao, China), which had excellent adhesive durability. The main properties of the two materials are listed in Table 1.

**Figure 3:**
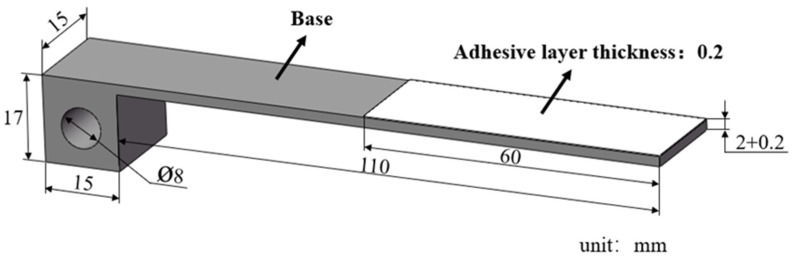
Geometry of double cantilever beam specimen.

**Figure 4:**
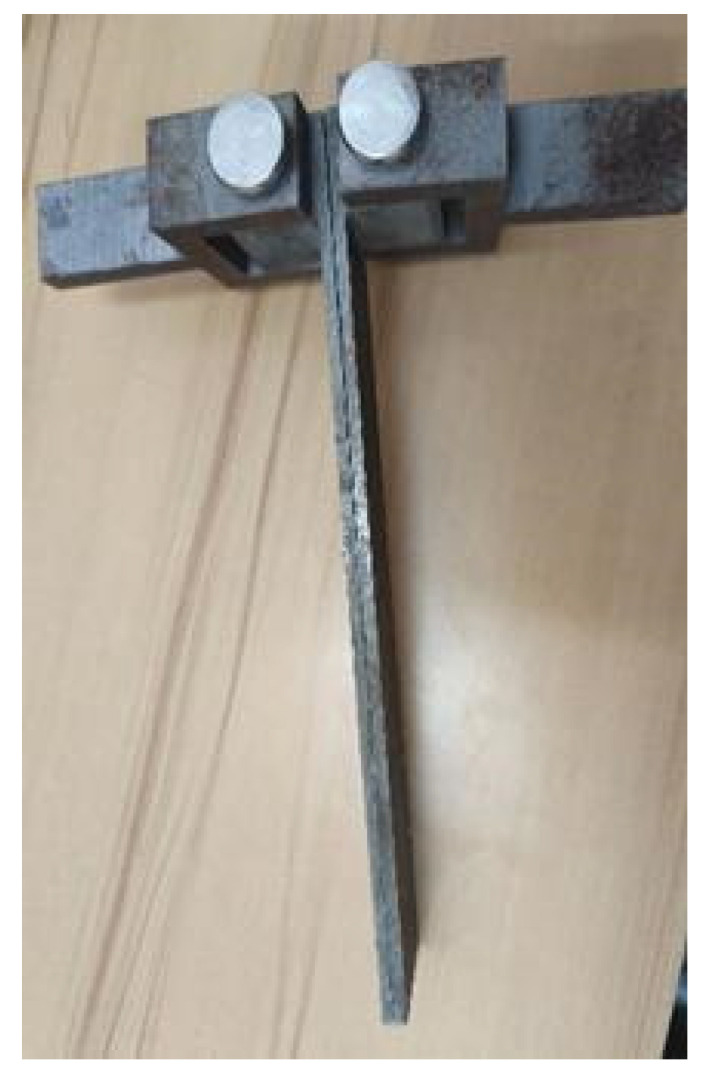
Physical photograph of the specimen.

According to the ASTM D5528 standard [37], a double cantilever beam (DCB) specimen was prepared, which was composed of upper and lower steel substrates bonded with an intermediate hot-melt adhesive layer. The shape and dimensional parameters of the DCB specimen are shown in Figure 3, and the steel substrate was directly processed into an L-shape, with a cube featuring a pin hole at its end. This design allowed it to be clamped to the testing machine’s chuck via a latch. The intermediate hot-melt adhesive layer was prepared with adhesive resin masterbatch using a hot press machine, and a long piece of 0.2 mm thick polytetrafluoroethylene film was placed between the two steel substrates to control the length and thickness of the adhesive layer. The prepared specimens are shown in Figure 4.

**Figure 5:**
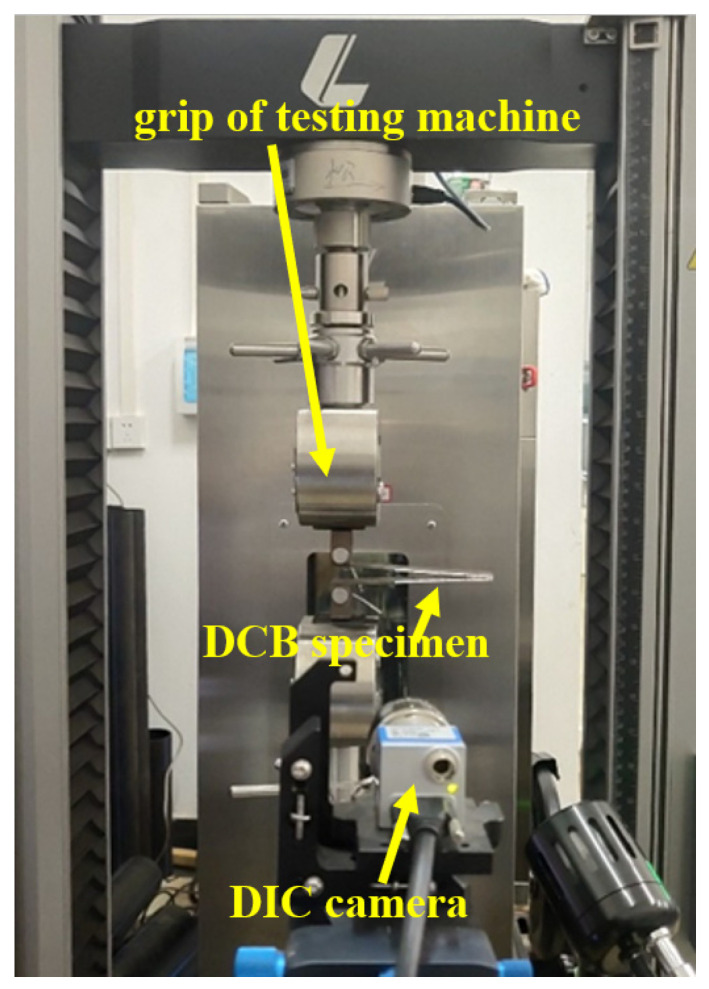
The DCB specimen under test.

The DCB specimens were clamped onto the universal tensile testing machine, as shown in Figure 5. The testing machine, connected to a computer, could conduct self-controlled tests. Because the instantaneous occurrence of crack propagation was difficult to observe and record, a Digital Image Correlation (DIC) system was used. The DIC camera simultaneously took synchronous photos of crack propagation when loading started, with a photo rate of 1 frame per second. All tests were conducted at room temperature. The testing method used displacement-controlled loading, with a tensile rate of 1 mm/min.

### 2.2. Test Results

**Figure 6:**
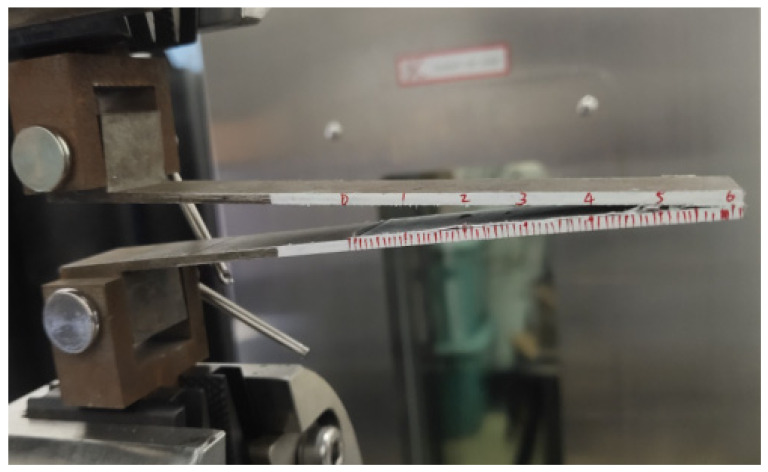
Interfacial debonding in the DCB specimen.

With displacement-controlled loading, the upper and lower substrates gradually separated, and the crack extended to a certain position. The force instantaneously decayed, indicating the end of the test, that is, the complete failure of the specimen, as shown in Figure 6.

**Figure 7:**
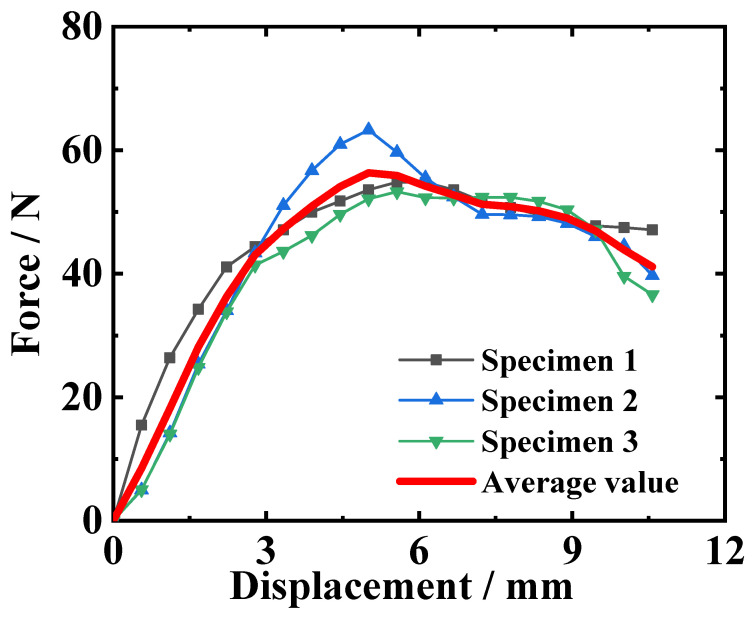
Reaction force–loading displacement curves of DCB tests.

Three DCB test curves are shown in Figure 7. The overall trends of several curves are consistent, but there is a certain degree of deviation between the curves. This is because in the sample preparation process, it is difficult to maintain consistency in surface roughness, adhesive thickness, and processability for each group of samples.

The reaction force and displacement values at the peak point of the reaction force–displacement curve are listed in Table 2. And the mean values of the maximum forces and the corresponding displacements are calculated for the following inversion analysis.

According to ASTM D5528 [37], the classical beam theory method is used here to preliminarily obtain the initial value of fracture energy, which can avoid the tediousness of repeated calculations necessitated by the wide range of interface parameter values. The beam theory expression for the strain energy release rate of an ideal double cantilever beam is given in Equation (1).

(1) $$G_{IC}=\frac{3P\delta }{2Ba}$$

$G_{IC}$ represents the fracture energy of mode I cracking (kJ/m^2^). *P* is the load (N), and δ is the load point deflection (mm). *B* is the width of the DCB specimen (mm). *a* is the delamination length (mm). Then, the peak load value and its corresponding load point deflection and delamination length are substituted into the equation, and the reference value of fracture energy is calculated, as listed in Table 3. The average value of the fracture energy is 0.44 kJ/m^2^, and this value can provide a reference for the selection of the initial value of the fracture energy in the inversion calculation.

## 3. Numerical Simulation

### 3.1. Cohesive Zone Model

**Figure 8:**
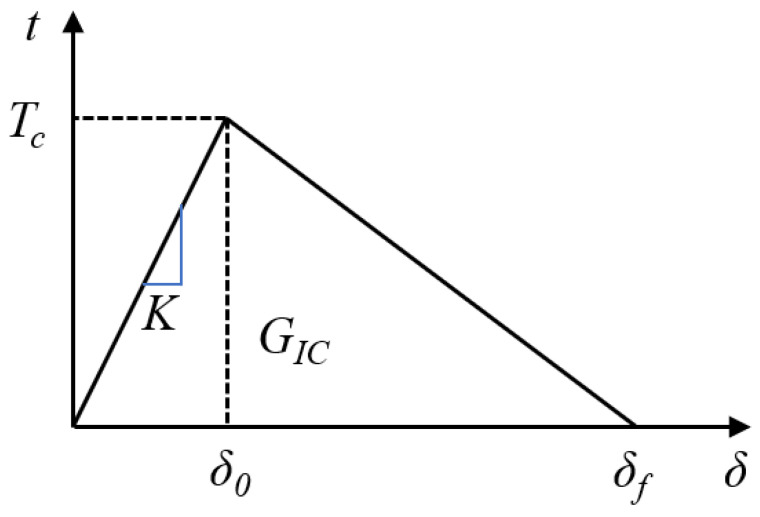
The schematic drawing of the bilinear cohesive zone model.

The cohesive zone model was used to simulate the interfacial debonding process. When simulating the process of crack propagation, the cohesive zone model did not require pre-existing cracks and was less dependent on finite element meshes. Common cohesive zone models included bilinear, trilinear, and parabolic models, among which the bilinear cohesive zone model was the most widely used. Because the shape of the cohesive zone model did not fundamentally affect the analysis results, this paper adopted the most commonly used bilinear cohesive zone model (as shown in Figure 8) for modeling and analysis of crack propagation processes.

In Figure 8, *t* is the interface stress, *δ* is the separation displacement of the interface, *T_c_* is the fracture strength at which the maximum interface stress is reached, *δ_0_* represents the separation displacement corresponding to reaching the fracture strength, *δ_f_* is the failure displacement, *K* is the interface stiffness, and *G_Ic_* is the normal fracture energy at the interface.

At the beginning, the interface stress *t* within the cohesive zone at the crack tip increases linearly with separation displacement *δ* under loading, marking the initiation stage of damage; when the interface stress *t* reaches its maximum value, corresponding to the fracture strength *T_c_* (*t* = *T_c_*), damage begins, and the cohesive zone at that point begins to evolve; thereafter, as the separation displacement *δ* increases, the interface stress *t* gradually decreases, leading to further crack propagation, marking the stage of damage evolution until the interface stress decreases to 0 and the crack at that point fully opens *δ* = *δ_f_*, indicating complete interface failure.

The damage initiation criterion in the elastic stage is represented by the elastic constitutive matrix.

(2) $$t=\left\{\begin{array}{c} t_{n}\\ t_{s}\\ t_{t} \end{array}\right\}=\left[\begin{array}{ccc} K_{nn} & K_{ns} & K_{nt}\\ K_{ns} & K_{ss} & K_{st}\\ K_{nt} & K_{st} & K_{tt} \end{array}\right]\left\{\begin{array}{c} \delta_{n}\\ \delta_{s}\\ \delta_{t} \end{array}\right\}=K\delta$$

Interface damage is assessed using the maximum nominal stress failure criterion. When defining material properties, a fracture damage propagation criterion is applied to the adhesive layer material. This is used to simulate damage initiation and the propagation of cracks in the adhesive layer. Damage begins when the maximum nominal stress ratio reaches a value of 1. This criterion can be expressed as

(3) $$\max\left\{\frac{\left\langle t_{n}\right\rangle }{t_{n}^{0}},\frac{\left|t_{s}\right|}{t_{s}^{0}},\frac{\left|t_{t}\right|}{t_{t}^{0}}\right\}=1$$

*t* represents interfacial stress, superscript 0 denotes fracture strength, and *n*, *s*, and *t*, respectively, represent the normal interface and two shear directions. In the equation, the Macauley brackets 〈〉 denote the meaning of

(4) $$\left\langle X\right\rangle =\left\{\begin{array}{cc} X, & X\geq 0\\ 0, & X<0 \end{array}\right.$$

〈*t_n_*〉 indicates that pure compressive stress does not cause damage.

### 3.2. Finite Element Model

**Figure 9:**
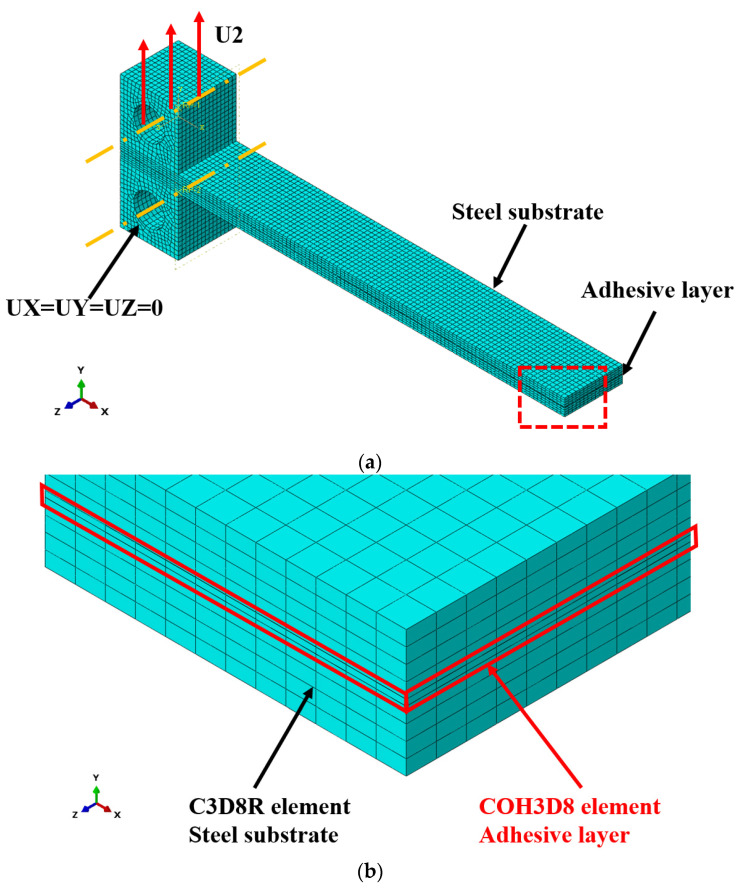
Finite element model of DCB specimen under tensile loading. (**a**) General view of model. (**b**) Enlarged view of model.

This paper focuses on investigating the cohesive parameters of steel and hot-melt adhesive in Type I opening damage. Therefore, the finite element software ABAQUS 2019 was employed to establish a three-dimensional DCB model for numerical simulation. The geometric shape and dimensional parameters of the model were consistent with the specimens used in the experiments. The base steel plate, Grade 45, was modeled using linear three-dimensional stress elements C3D8R, while the adhesive layer adopted cohesive elements COH3D8, with an initial thickness of 0.2 mm. It is worth noting here that C3D8R elements were prone to shear locking and might introduce spurious stiffness in bending-dominated problems when discretization across the thickness is insufficient. This effect could artificially increase the apparent flexural stiffness of the beams, potentially influencing the accuracy of the simulated load–displacement response. Hence, the steel plates were meshed into 4 layers to provide enough discretization across the thickness, which was verified by the authors by comparing the simulation results between two models with C3D8R and C3D20R elements. After meshing was conducted, displacement loading was utilized, and the boundary conditions are illustrated in Figure 9a. The element types of the substrates and the adhesive layer were shown in Figure 9b, which was a local magnification of the area enclosed by the red dashed box in Figure 9a.

In the following, mesh sensitivity is analyzed, and the result is listed in Table 4. It can be found that when the mesh number is 506,100, the peak force of the DCB specimen under tensile loading is 57.91 N, and this result is relatively accurate. To analyze the mesh sensitivity of the model, different element sizes are employed, and various element numbers are obtained. To facilitate the comparison of model calculation results, the calculated values of each peak reaction force are normalized with reference to the calculation result of the model with the maximum number of meshes, as shown in Table 4. It can be observed that as the mesh size increases, the number of meshes decreases, and the calculated maximum reaction force of the model gradually decreases. However, the calculation results of all models are relatively close. Ultimately, considering the balance between computational cost and accuracy, the mesh size of the model in this paper was set to 1 mm, and the element number of the model was 20,910.

### 3.3. Inversion Method

**Figure 10:**
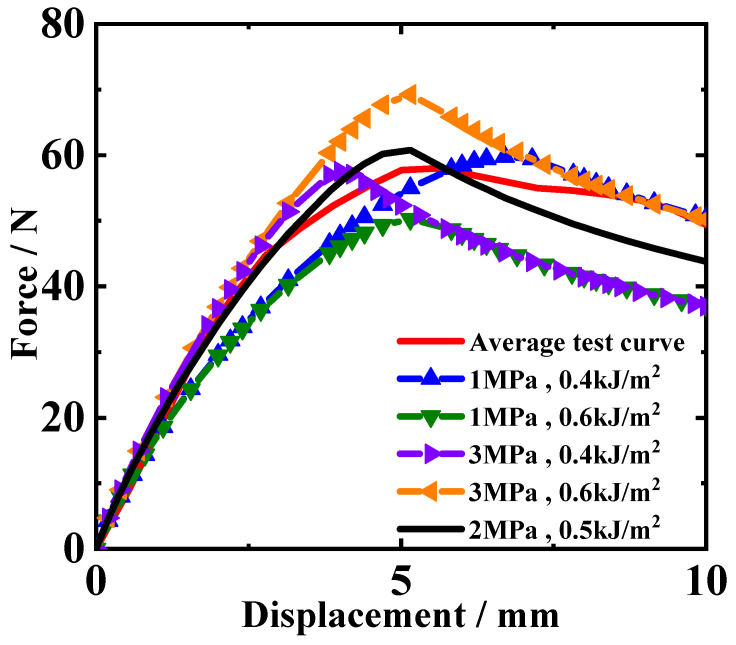
The influence of interfacial parameters on the trend of the DCB reaction force–loading displacement curve.

In the cohesive zone model, there are two crucial parameters, including interfacial strength and fracture energy. Both of these two values are quite difficult to directly measure through experimental testing. To overcome the challenge of obtaining interfacial parameters, this study employs an inverse calculation method and develops a program to execute the inverse calculation. The inverse calculation program essentially constitutes an optimization procedure, continually iteratively adjusting key parameters of the DCB finite element model to optimize the simulated result and make it gradually match the experimental results. When the simulated result sufficiently matches the experimental results, the interfacial parameters in the finite element model can be considered the true solution to characterize the interfacial mechanical behavior. After many repeated iterations, the calculation result curves are closer and closer to the experimental result curves. Then, when the matching degree of the two results meets a certain condition, the interfacial parameters are determined. Thus, the core algorithm underlying this inversion process is an optimization algorithm, and this study selects the pattern search method for optimization calculations. The matching degree of the simulated and experimental results is represented by the consistency of the reaction force–loading displacement curves from simulated and test results. By pre-setting different interfacial parameter values in the DCB model, it is known from Figure 10 that the trend of the simulated reaction force–loading displacement curve mainly depends on the peak value of the reaction force and its corresponding displacement.

As seen from the curves in Figure 10, the change in interface parameters has little impact on the trend and shape of the curves, but it can determine the peak point of the curve. Therefore, the inversion objective function only needs to focus on the peak point. When making the peak point of the simulated result curve consistent with that of the experimental curve, the purpose of inversion calculation can be achieved. Thus, the objective function of the inversion calculation is constructed around the peak point, as in Equation (5):

(5) $$R=\sqrt{{\left(P_{sim}^{peak}-P_{exp}^{peak}\right)}^{2}}+\sqrt{{\left(S_{sim}^{peak}-S_{exp}^{peak}\right)}^{2}}$$

In the equation above, *P* stands for the load, *S* stands for the displacement, *sim* denotes the simulated results, *exp* indicates the experimental results, and the superscript *peak* signifies the values corresponding to the point of the peak load in the load–displacement curve.

In addition, the influence of interfacial parameters on the curve’s trend can help determine the variation range of interfacial parameters. In Figure 10, an average test curve is obtained by averaging the three test curves, as shown in Figure 7, and this curve is used as a reference for the parameter influence analysis. According to the simulated results of models with different interfacial parameters, it can be found that the interfacial strength only affects the slope and peak load of the rising segment. On the other hand, the fracture energy determines the descending part of the curve. From Figure 10, it can be observed that when interfacial strength is 3 MPa and fracture energy is between 0.4 kJ/m^2^ and 0.6 kJ/m^2^, the descending segment of the experimental curve might lie within this range. Therefore, the interfacial strength possibly ranges from 1 to 3 MPa, and the fracture energy possibly ranges from 0.4 to 0.6 kJ/m^2^. As for the interfacial strength, the range is set between 5 MPa and 20 MPa. The initial values for inversion parameters are set to 10 MPa for interfacial strength and 0.4 kJ/m^2^ for fracture energy.

**Figure 11:**
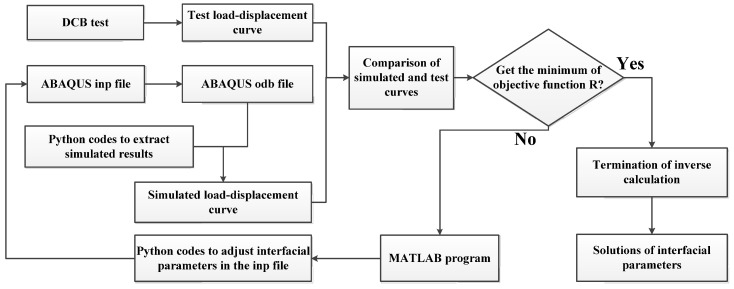
Flowchart of inversion calculation.

The flowchart of the inversion calculation is shown in Figure 11. The inverse optimization process is entirely conducted in MATLAB (2022 version), operating automatically without requiring manual intervention. It utilizes a main MATLAB (2022 version) program to call a Python (2.7 version) program to modify the ENERGY and MAXS data in the inp file, followed by invoking MATLAB (2022 version) subprograms for ABAQUS (2019 version) finite element calculations. Finally, modification and extraction of finite element input and output data are achieved through MATLAB (2022 version) by calling Python (2.7 version) programs.

## 4. Results and Discussion

### 4.1. Inversion Results

**Figure 12:**
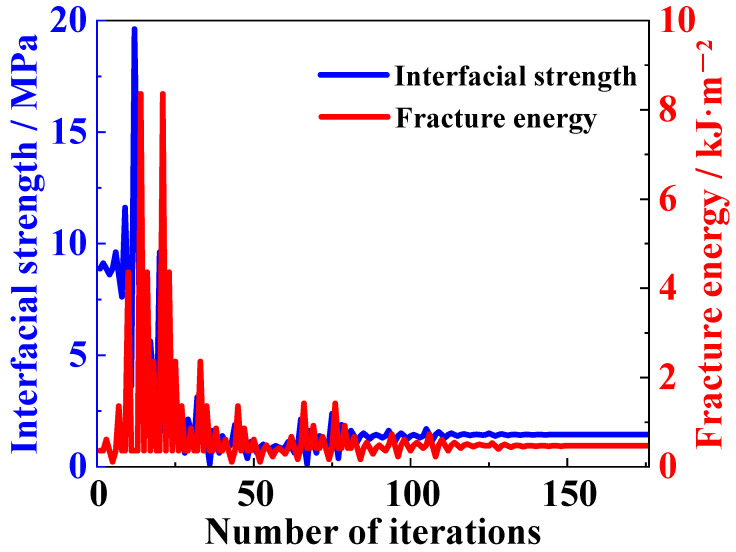
Variation of interfacial parameters during the iterative process of inversion calculation.

The inversion parameter optimization process of the finite element model is illustrated in Figure 12. The variation trend of interface shear strength and fracture energy during the inversion iterations resembles two zigzagging lines. The graph indicates that parameter optimization generally stabilizes after around 116 iterations, suggesting that the convergence values of the inversion parameters have been largely determined. However, it is after 148 iterations that the inversion parameters finally cease to change. The duration of the inversion process is around 17 h, the calculated interface strength is 1.44 MPa, and the interfacial fracture energy is 0.47 kJ/m^2^.

**Figure 13:**
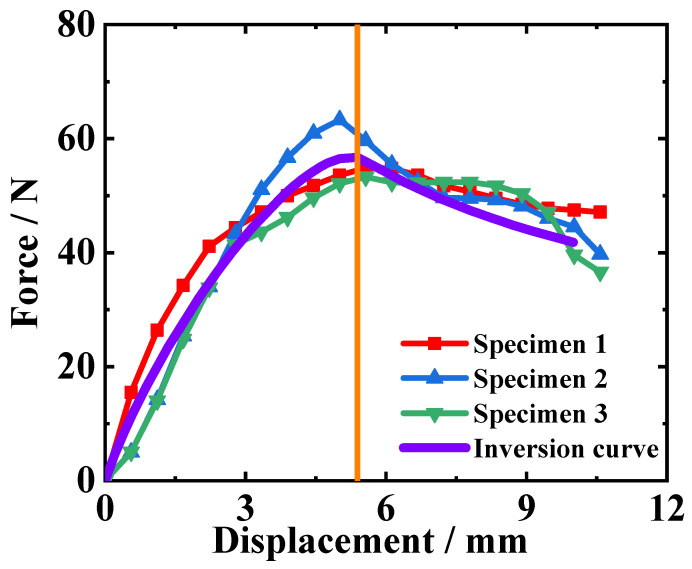
Comparison of DCB simulation curve and test curves; the orange vertical line marks the moment when simulated crack tip propagation starts to occur.

Substituting the inversion results of the interfacial parameters into the DCB model, the simulation reaction force–loading displacement curve is shown in Figure 13. The simulated curve matches the experimental curves. However, the simulation model does not consider the influence of the curing temperature of the adhesive layer on its fracture characteristics, and the simulation neglects the micro-scale defects. These factors may be causing deviation between the simulated and experimental curves. Comparing the data obtained from the simulation and the experiment (Table 2), the maximum load in the DCB simulation is 56.72 N, corresponding to a displacement of 5.39 mm, while the average maximum load in the experiment is 57.14 N, corresponding to an average displacement of 5.38 mm. The relative error of the maximum load is −0.74%, and the relative error of the corresponding displacement is 0.19%. This indicates that the inversion calculation method is correct and effective, and the finite element results based on the inversion parameters are consistent with the DCB test results.

### 4.2. Interfacial Failure Process

**Figure 14:**
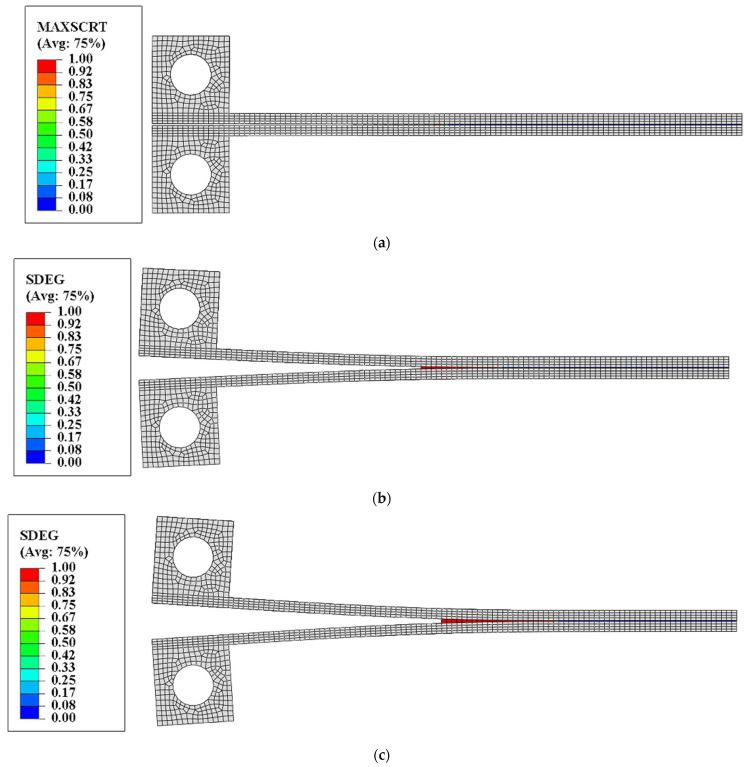
Interfacial debonding process of the DCB specimen model. (**a**) Damage occurs at the interface when the loading displacement is 0.2 mm. (**b**) Complete damage of a cohesive element occurs when the loading displacement is 3.825 mm. (**c**) Crack propagation occurs when the loading displacement is 5.39 mm.

As shown in Figure 14, contour plots of the interfacial damage corresponding to different increment steps are presented. In the first increment step, the maximum nominal stress criterion is met for the adhesive interface when the vertical loading displacement is 0.2 mm, and the MAXSCRT reaches one, indicating that damage starts to occur at the interface. Meanwhile, all of the SDEG values of the interface elements are still less than one, indicating that the interface elements have not yet reached the fully damaged state (complete stiffness degradation), and no macroscopic crack propagation has occurred at the interface at this point. As the loading displacement gradually increases to 3.825 mm, the SDEG of the interface elements at the crack tip starts to reach one. At the same time, the reaction force–displacement curve is not reaching its peak point. After that, as the loading displacement continues to increase, some cohesive elements are deleted, and the macroscopic cracks gradually propagate. This moment is represented by the orange vertical line shown in Figure 13. After that, the macroscopic cracks gradually propagate, and, accordingly, the curve begins to slowly decrease. In the following, as the opening of the DCB specimen increases, more and more interface elements are deleted, and the macroscopic crack propagation can be observed in the model.

### 4.3. Crack Verification

**Figure 15:**
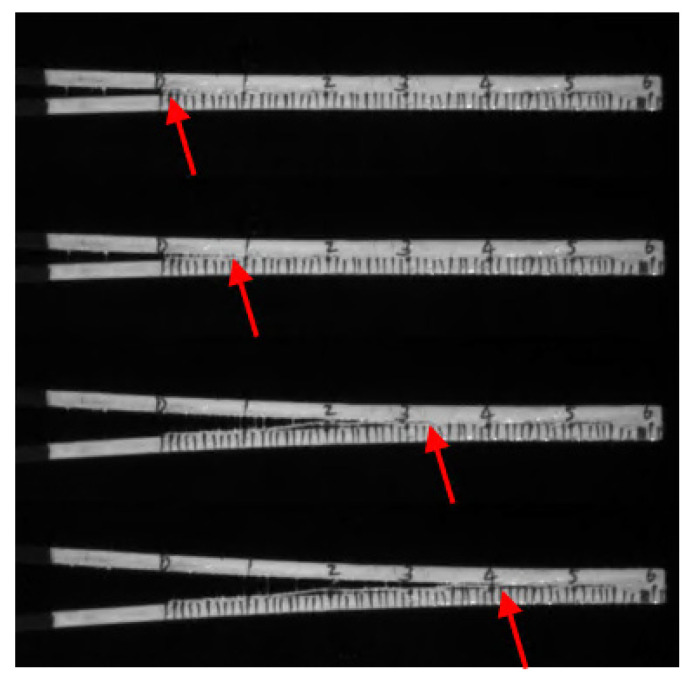
Crack length acquired using DIC camera. Red arrows indicate the location of crack tip.

In the DCB test, not only can the reaction force–displacement curve be obtained but also the curve of the crack tip propagation displacement with the loading displacement, as shown in Figure 15. To observe the pattern of crack propagation, the images captured using the DIC camera are analyzed frame by frame. The crack length corresponding to each time point is obtained based on the scale markings on the side of the specimen; therefore, curves of crack length versus loading displacement can be made for multiple specimens.

**Figure 16:**
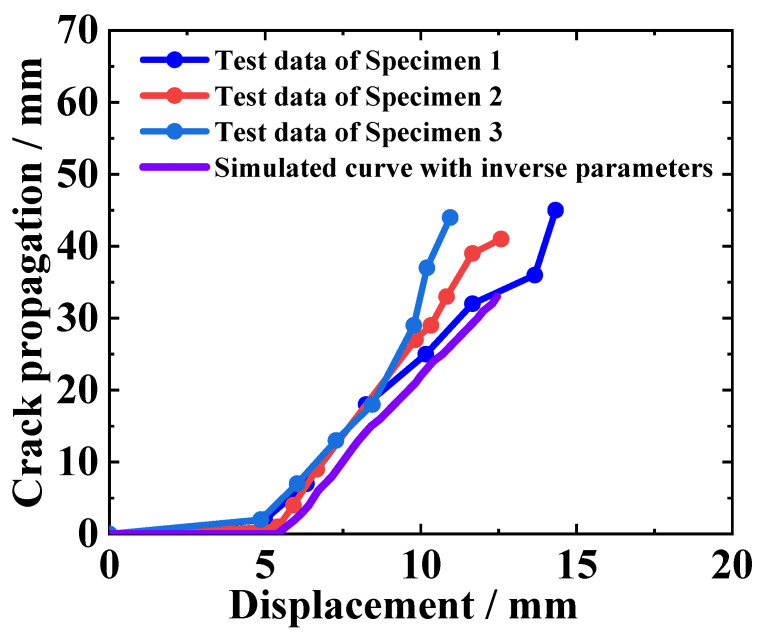
Comparison of crack tip propagation between simulated and test results.

By substituting the parameters obtained from inversion into the finite element model, the propagation process of the crack tip with the loading displacement can be reproduced through the DCB finite element model. Figure 16 shows that the curve of the finite element simulation results for the crack tip propagation process is basically consistent with the trend of the crack propagation curves of the three specimens. This proves the effectiveness of the inversion calculation method from another dimension, indicating that the simulation results of the DCB model are consistent with the experiment.

## 5. Conclusions

(1) Double cantilever beam (DCB) specimens were prepared using steel strips and hot-melt adhesive to investigate interfacial failure behavior. A Digital Image Correlation (DIC) system was set up to monitor crack tip propagation. After the tests, the loading curve of reaction force versus loading displacement could be obtained, as well as the curve of crack tip propagation versus loading displacement. The former is used for the inverse calculation of interfacial parameters, while the latter is used to verify the accuracy and reliability of the obtained interfacial parameters from a new dimension.
(2) A finite element model was established to represent the DCB specimen under tensile loading. Cohesive elements were employed to characterize the steel/polymer interface. As the two key cohesive parameters, interface strength and fracture energy, could not be directly obtained, an inversion optimization calculation method was thus proposed to determine the interfacial parameters. An ABAQUS–Python–MATLAB interactive program was developed to employ the pattern search algorithm and adjust the key interfacial parameters during iterative calculations. As expected, in the iterative calculations round by round, the program successfully made the computed load–displacement curve increasingly close to the experimental curve. Finally, the program met the convergence criteria, and the interface parameters were solved.
(3) Furthermore, crack tip propagation with loading displacement was employed to verify the validity of the inversion calculation method. By substituting the parameters obtained from inversion into the finite element model, the propagation process of the crack tip with loading displacement could be reproduced through the DCB finite element model. It showed that the curve of the finite element simulation results for the crack tip propagation process was basically consistent with the trend of the crack propagation curves of the three specimens. This proved the effectiveness of the inversion calculation method from another dimension, indicating that the simulation results of the DCB model were consistent with the experiment.

The inversion calculation method can effectively help determine the interfacial parameters of mode I debonding, which provides important basic data for establishing the interfacial failure criterion of the steel–plastic interface in the PSP. Moreover, this research could be helpful for investigations on mode I debonding of various composite structures. In the future, mode II/III debonding and mixed-mode cracking should be studied to fully represent the practical interfacial failure behavior of the PSP or other similar composite structures.

## Figures and Tables

**Table 1:** Material properties of steel and hot-melt adhesive.

Material	Density (g/cm^3^)	Elastic Modulus (MPa)	Yield Strength	Poisson’s Ratio
45# steel	7.8	204,309	284	0.26
Hot-melt adhesive	0.95	625	\	0.45

**Table 2:** The reaction force and displacement values at the peak point of the reaction force–displacement curve.

Item	Maximum Load (N)	Corresponding Displacement (mm)
Specimen 1	54.84	5.56
Specimen 2	63.30	5.01
Specimen 3	53.28	5.57
Mean value	57.14	5.38

**Table 3:** Calculated fracture energy based on beam theory expression using DCB test data.

Item	Fracture Energy (kJ/m^2^)
Specimen 1	0.44
Specimen 2	0.45
Specimen 3	0.42
Average	0.44

**Table 4:** Simulated results of the DCB models with different element sizes and numbers.

Element Size (mm)	Element Number	Peak Reaction Force (N)	Normalized Value
2	4952	56.64	0.978
1	20,910	56.72	0.979
0.5	106,260	56.79	0.981
0.3	506,100	57.91	1

## Data Availability

The original contributions presented in this study are included in the article. Further inquiries can be directed to the corresponding author.
